# Identification of DNA methylation patterns predisposing for an efficient response to BCG vaccination in healthy BCG-naïve subjects

**DOI:** 10.1080/15592294.2019.1603963

**Published:** 2019-04-22

**Authors:** Jyotirmoy Das, Deepti Verma, Mika Gustafsson, Maria Lerm

**Affiliations:** a Department of Clinical and Experimental Medicine (IKE), Faculty of Medicine and Health Sciences, Linköping University, Linköping, Sweden; b Department of Clinical and Experimental Medicine (IKE), Division of Cell Biology (CELLB), Linköping University, Linköping, Sweden; c Department of Physics, Chemistry and Biology (IFM) Bioinformatics (BION), Linköping University, Linköping, Sweden

**Keywords:** DNA methylation, BCG-vaccination, phagocytosis, actin regulation, Mycobacterium tuberculosis, Tuberculosis

## Abstract

The protection against tuberculosis induced by the Bacille Calmette Guérin (BCG) vaccine is unpredictable. In our previous study, altered DNA methylation pattern in peripheral blood mononuclear cells (PBMCs) in response to BCG was observed in a subgroup of individuals, whose macrophages killed mycobacteria effectively (‘responders’). These macrophages also showed production of Interleukin-1β (IL-1β) in response to mycobacterial stimuli before vaccination. Here, we hypothesized that the propensity to respond to the BCG vaccine is reflected in the DNA methylome. We mapped the differentially methylated genes (DMGs) in PBMCs isolated from responders/non-responders at the time point before vaccination aiming to identify possible predictors of BCG responsiveness. We identified 43 DMGs and subsequent bioinformatic analyses showed that these were enriched for actin-modulating pathways, predicting differences in phagocytosis. This could be validated by experiments showing that phagocytosis of mycobacteria, which is an event preceding mycobacteria-induced IL-1β production, was strongly correlated with the DMG pattern.

## Introduction

DNA methylation is one of the most studied epigenetic modifications in humans, playing a critical role in cellular response, development and differentiation [1,2]. Recent studies show that interaction of host cells with microbial components evokes modulation at the epigenomic level in the host cells. Such epigenomic reorganization can be both protective (enhancing host defence and inducing trained immunity, reviewed in [3,4]) and harmful (dysregulating host functions and facilitating the survival of the pathogen, reviewed in [5]). Tuberculosis (TB) is an infectious disease caused by *Mycobacterium tuberculosis*, which invades human macrophages and employs several mechanisms to manipulate macrophage anti-microbial functions [6]. Still, provided that cellular defence mechanisms are active, for example, a functional interferon-γ signalling system [7,8], and the bacterial load is limited, *M. tuberculosis* replication can be controlled by macrophages [9,10] Also, genetic variants causing enhanced interleukin (IL)-1β production can contribute to the capacity of macrophages to restrict mycobacterial growth [11,12]. In a previous study [7], we observed that macrophages from a subset of subjects vaccinated with the TB vaccine, Bacille Calmette-Guérin (BCG), displayed an enhanced anti-mycobacterial response. Following vaccine administration, the immune cells isolated from these ‘responders’ concomitantly showed an altered DNA methylation pattern enriched in immune pathways, suggesting the induction of trained immunity. In responders, we also observed the effective production of IL-1β in response to mycobacterial stimuli of macrophages *at the time point before* BCG vaccination, suggesting that responders’ immune cells were pre-conditioned to mount a proper response towards mycobacteria. In order to investigate whether this pre-conditioned responsiveness was reflected in differing DNA methylation patterns in responders’ and non-responders’ immune cells, we reanalysed the DNA methylome data from this time point, including the whole dataset and not just the transcription start site as was the case in the previous study. When comparing responders and non-responders we identified 43 genes with a unique methylation pattern in the responder group. Via pathway analyses and module discovery, these genes were found to be enriched for actin-reorganization, a process crucial for phagocytic uptake of bacteria [13]. Guided by this observation, our data also revealed that phagocytosis of mycobacteria was significantly higher in macrophages obtained from responders (‘primed macrophages’) as compared to non-responders (naïve macrophages). Finally, using multifactorial analyses, we confirmed the strong correlation between the identified DMGs and phagocytic uptake of mycobacteria, whereas the stronger IL-1β production in primed macrophages is likely to be secondary to the observed phagocytosis efficacy.

## Results

### Identification of 43 genes with differential methylation patterns comparing data from BCG-naïve responders and non-responders

**Figure 1.**
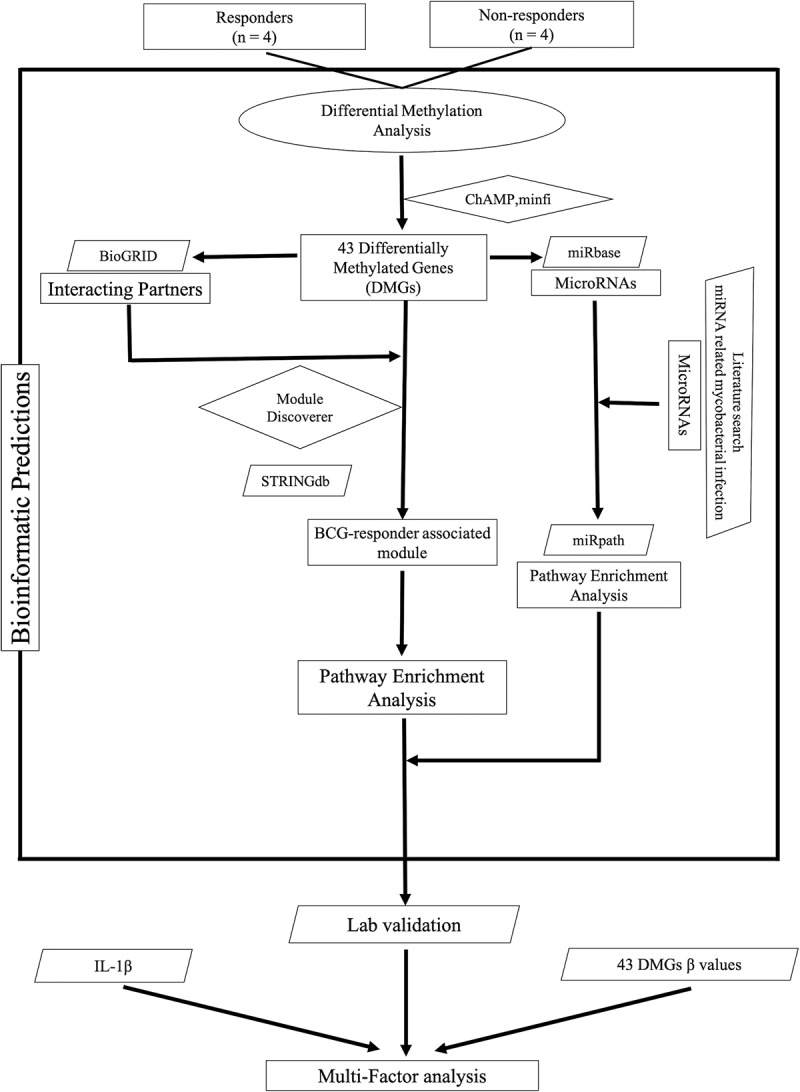
Flow chart of the data analysis approach used in this study (ellipses represent start/end point (source data), parallelograms represent input data (used databases), diamonds represent source codes (R scripts) and rectangles represent steps (results/outputs).

**Figure 2.**
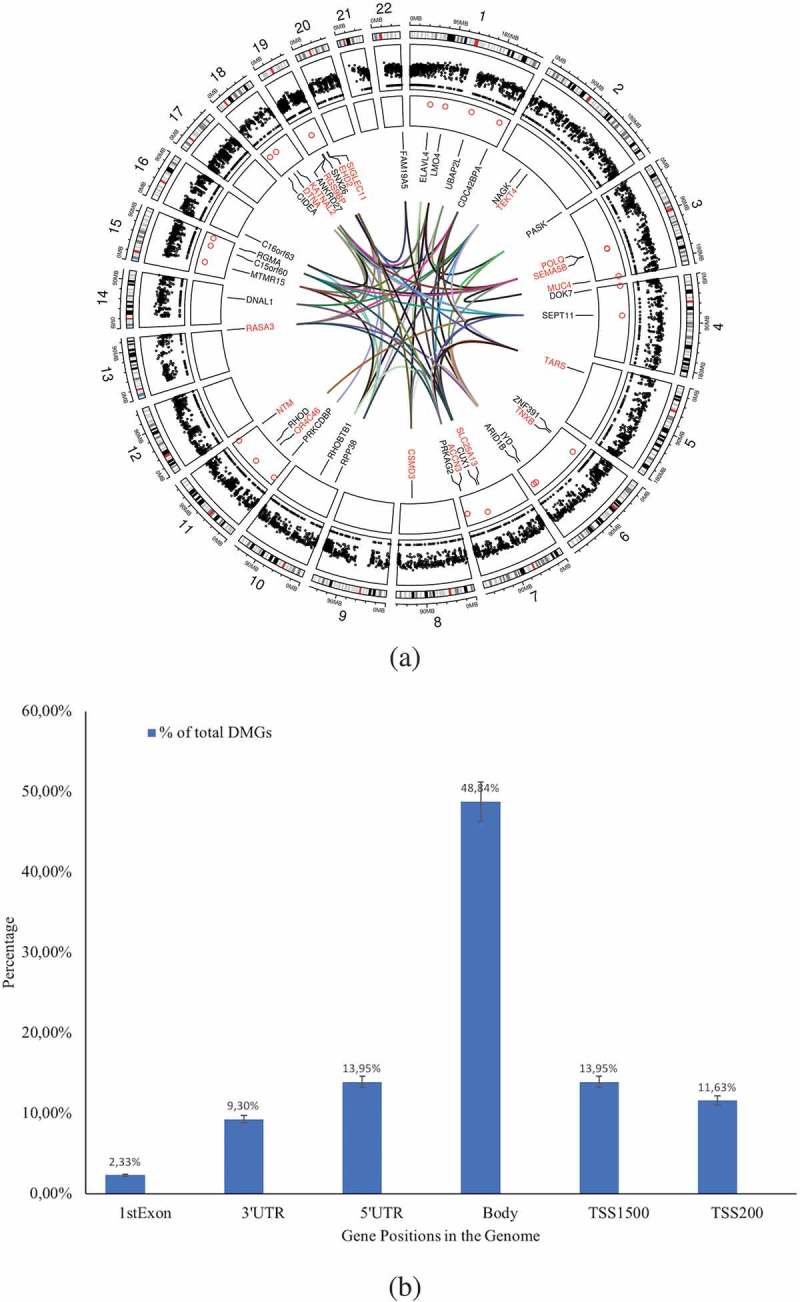
(a) Circos plot of the DMGs’chromosomal location and interaction generated using Circos (Circlize package in R v3.4). The outer circle reflects the 22 autosomes (the scale represents the chromosomal location in megabases(MB) and the circle shows the cytoband colours used in UCSC hg19 data sources (using the default parameter in ‘circlize’ package), the middle circle in black dots reflects the 414,206 Illumina probes after filtering, the inner circle indicates the chromosomal location of the 43 DMGs. The interconnections among the genes (identified using GeneMania package in Cytoscape v3.5) is shown by coloured lines. Blue: physical interactions, pink: genetic interactions, green: co-expression, violet: co-localizations among DMGs. The strengths of the interactions are shown by shades of colour from light (weak) to deep (strong). (b) Percentage of the DMGs (out of total DMGs) with altered DNA methylation pattern in the genomic positions 1^st^ Exon, 3’UTR, 5’UTR, gene body, TSS1500, and TSS200.

Our previous observation that traits such as an effective release of IL-1β in response to mycobacterial infection existed in subjects who later responded to the BCG vaccine [7] urged us to investigate whether the responders’ immune cells exhibited a distinct DNA methylation pattern prior to BCG vaccination. To this end, we performed a thorough analysis of the whole DNA methylation data set obtained from immune cells collected at the time point before the BCG vaccine was administered. Our strategy was to first identify differences in methylation patterns between the responder and non-responder groups and then perform two parallel analyses of interacting neighbours and miRNAs, performing module search and identifying pathways that could guide experimental exploration of suggested mechanisms (Figure 1). Following this approach, we were able to identify 43 genes displaying a differential methylation pattern when comparing responders and non-responders (*p*-value < 0.05 and BH corrected *p*-value < 0.05) (Figure 2(a) & Supplementary Table 1). Among these 43 differentially methylated genes (DMGs), 3 were hypo-methylated in the responder group (SNX26, encoding a GTPase-activating protein, EHD2 encoding a protein involved in membrane trafficking, and OR4C46 encoding an olfactory receptor). The majority of the differentially methylated sites were localized to the gene body (49%, Figure 2(b)).

### Pathway enrichment analysis of the DMGs reveals a BCG responder-associated module

To evaluate for significant pathway enrichment of the 43 DMGs, we first explored the gene–gene interaction data using BioGRID database v3.4 [14] and Cytoscape v3.5 [15].

**Figure 3.**
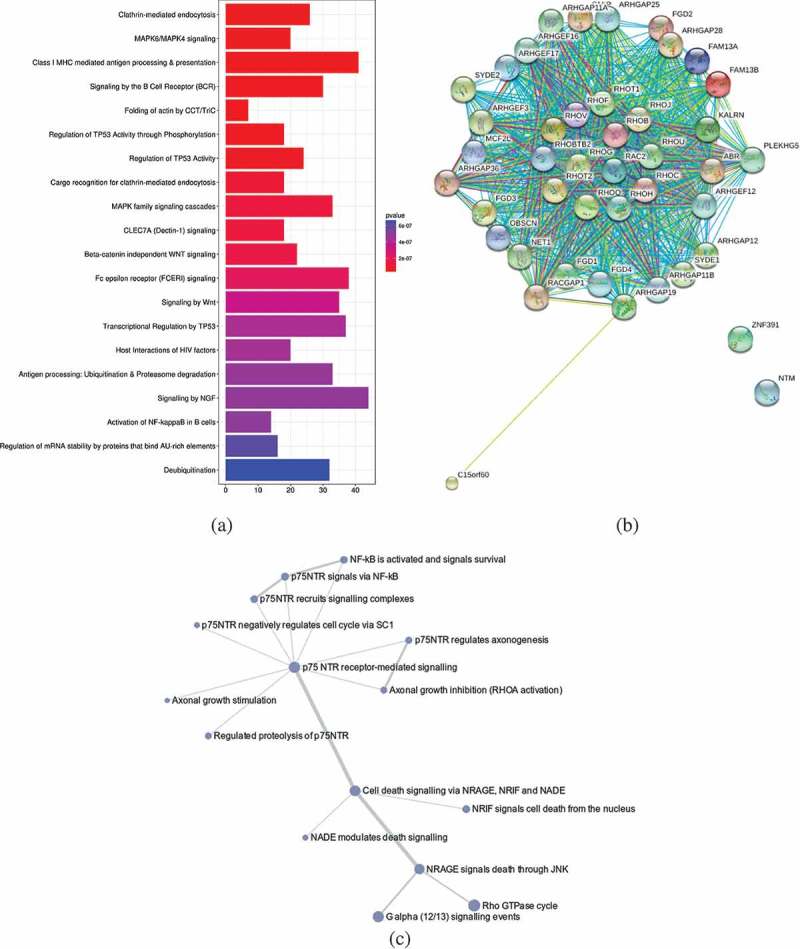
(a) The top 20 pathways identified by analysing the 43 DMGs and their first interacting gene partners using ReactomePA package in R. Enrichment based on Bonferroni-Hochberg adjusted *p*-values is shown in a colour scale of red (high enrichment) to blue (low enrichment). The length of the bar in the plot illustrates the enrichment score, reflecting the number of pathway partners in the database. Background score was calculated based on the number of genes of the functional pathway in the database to normalize the data. (b) Interaction map of a putative BCG responder-asociated module. The proteins expressed from the 43 DMGs and their first interacting neighbours (calculated from BioGrid database) were analysed using the ModuleDiscoverer package to identify the enriched cluster in the dataset, and portrayed in the STRINGdb package in R. With a score threshold cut-off of 900, the resulting network contained 651 interactions between 41 proteins; two of them (ZNF391 and NTM) without interactions. Nodes are coloured according to the log fold change ratio from red (high fold change) to green (low fold change). The overall interactions had the *p*-value≈0 calculated by the number of overall interactions over the number of expected interactions. (c). The of p75-NTR signalling network as identified from BCG responder-associated module using PathCards database. The nodes represent the associated pathways and the edges represent Jaccard similarity coefficient (J). The node size represents the distributed gene count across different sources (proportional to the number of interacting pathways), the edge length represents the proximity the neighbour, while the edge widths are proportional to the pairwise J computed for the gene contents of the entire source.

The DMGs and their first neighbour interacting partners are shown in Figure S1. We selected the interacting gene partners for each of the DMGs and detected their pathway enrichment using ReactomePA package (Figure 3(a)). We extracted the top 20 enriched pathways and observed that these pathways were mainly associated with immune system (six pathways), followed by signal transduction (five pathways), gene expression (three pathways), metabolism (three pathways), infectious disease (two pathways), and transport (one pathway). Importantly, among these pathways, we also observed the CLEC7A (Dectin-1) signalling pathway, which triggers phagocytosis and induces the production of IL-1β [16]. For functional analysis, the ModuleDiscoverer package in R (gene-based) [17] was linked to the STRINGdb to translate to the proteins corresponding to the DMGs [18]. With this approach, we identified a module of 43 proteins that were interacting with the DMGs. This putative BCG responder-associated module displayed 651 interactions (Figure 3(b)). The majority of the proteins observed in the module were associated with the p75 neurotrophin receptor (NTR)-mediated signalling network, which connects 15 pathways and is implicated in the regulation of actin reorganization [15,16] (Figure 3(c)).

### Justification of the BCG responder module by validation against other studies

**Figure 4.**
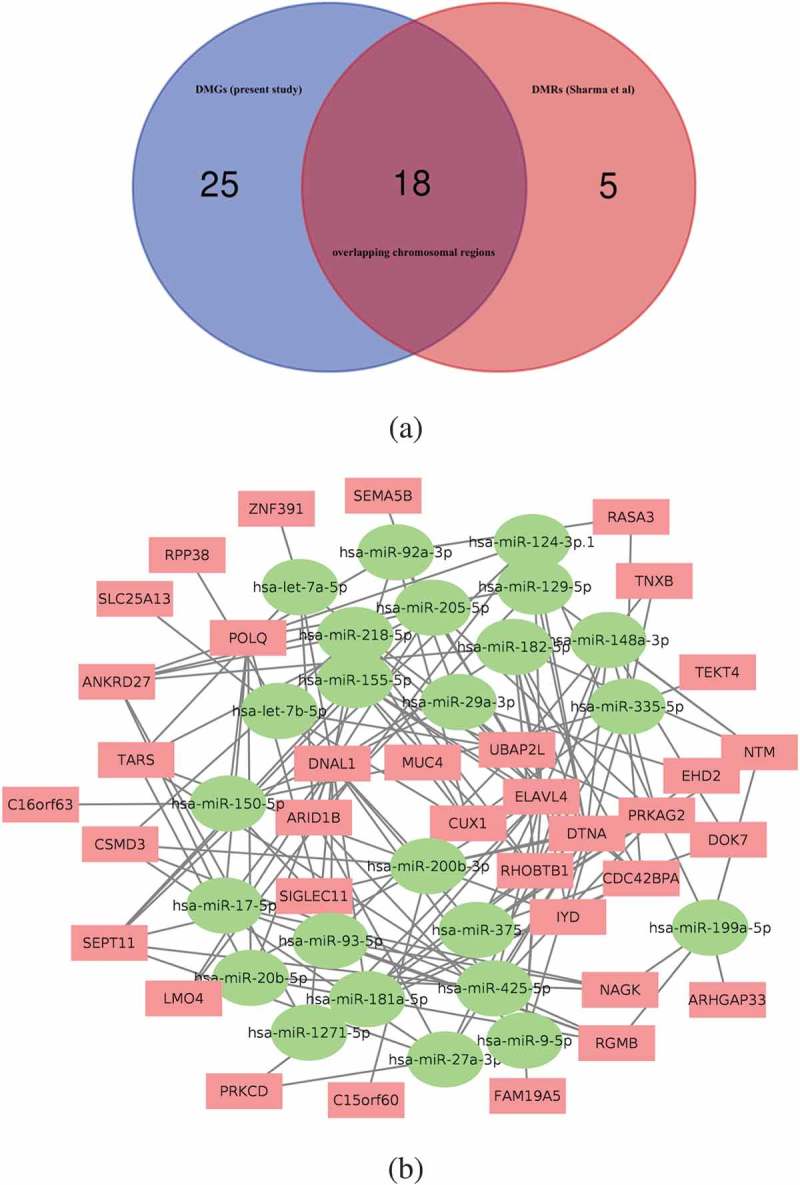
(a) Venn diagram showing the 18 genes overlapping their chromosomal positions after matching the 43 DMGs identified in the current study with the 23 DMRs reported in Sharma *et.al*. after 1,000,000 permutations in a randomization test. (b) Interaction network map based on the connection between 35 DMGs and 24 miRNAs that overlap between our DMG-interacting miRNAs and the previously published miRNAs linked to mycobacterial exposure of human cells or tissues. Red rectangular nodes show the DMGs and green circular nodes represent the interacting miRNAs. See also Supplementary Table 2.

Next, we sought to identify the general relevance for TB by comparing the identified DMGs with other studies on host genes targeted during the interaction between *M. tuberculosis* and human immunity. Sharma *et al*. [19] recently identified 23 regions in the DNA of human macrophages that displayed an altered methylation pattern in response to experimental infection with *M. tuberculosis*. We scrutinized the chromosomal locations of the 43 DMGs of our dataset and matched these with the 23 differentially methylated regions (DMRs) in Sharma’s study and performed an overlap enrichment analysis using a randomization test with 1,000,000 permutations. The result showed a statistically significant overlap of 18 of the 43 DMGs (*p-*value = 2.0 × 10^–3^) between the two datasets (Figure 4(a)).

Recent reports have shown differential expression of host microRNA (miRNA) in response to mycobacterial exposure [20–23]. Using a set of filtration methods, we retrieved information from several databases to identify a set of miRNAs that target the 43 DMGs. We identified a total of 496 unique miRNAs targeting the 43 DMGs (DMG-miRNA) with 791 connections, ranging from 1 to 8 targeted genes for each miRNA. For a robust selection of DMG-miRNAs, we further narrowed down the list to only include those miRNAs that target at least two DMGs, leaving a total of 129 unique DMG-interacting miRNAs for further analysis. In parallel to this, we also prepared a list of 313 miRNAs that have been linked to mycobacterial exposure of human cells or tissues in previously published literature [24,25]. Matching the DMG-miRNA with these mycobacteria-specific miRNAs, we found that 24 of the 129 DMG-miRNA were represented in the list of miRNAs related to mycobacterial exposure. Enrichment analysis assessing whether this overlap was higher than expected by chance resulted in an enrichment factor of 1.6 (*p*-value < 0.014). Furthermore, one-third of known miRNAs have been shown to have more than one target sequence [26], and the 24 overlapping miRNAs interacted with 35 out of the 43 DMGs in our dataset, further expanding the impact of the overlap. Figure 4(b) shows the network analysis of these 24 miRNA with the 35 DMGs. Next, we performed a pathway enrichment analysis with DIANA miRpath v3 database using these 24 miRNAs (Figure S2 and Supplementary Table 2). The most enriched pathway, the ECM-receptor interaction pathway, transmits biochemical signals and mechanical forces across the plasma membrane, thereby modulating the actin cytoskeleton [27]. Again, the neurotrophin signalling pathway, identical to the p75 NTR pathway identified in Figure 3(c) [28] was revealed in the analysis. Taken together, these two analyses independently support the high TB relevance of the BCG responder-associated module.

### Functional association of the BCG responder-associated module and phagocytosis

**Figure 5.**
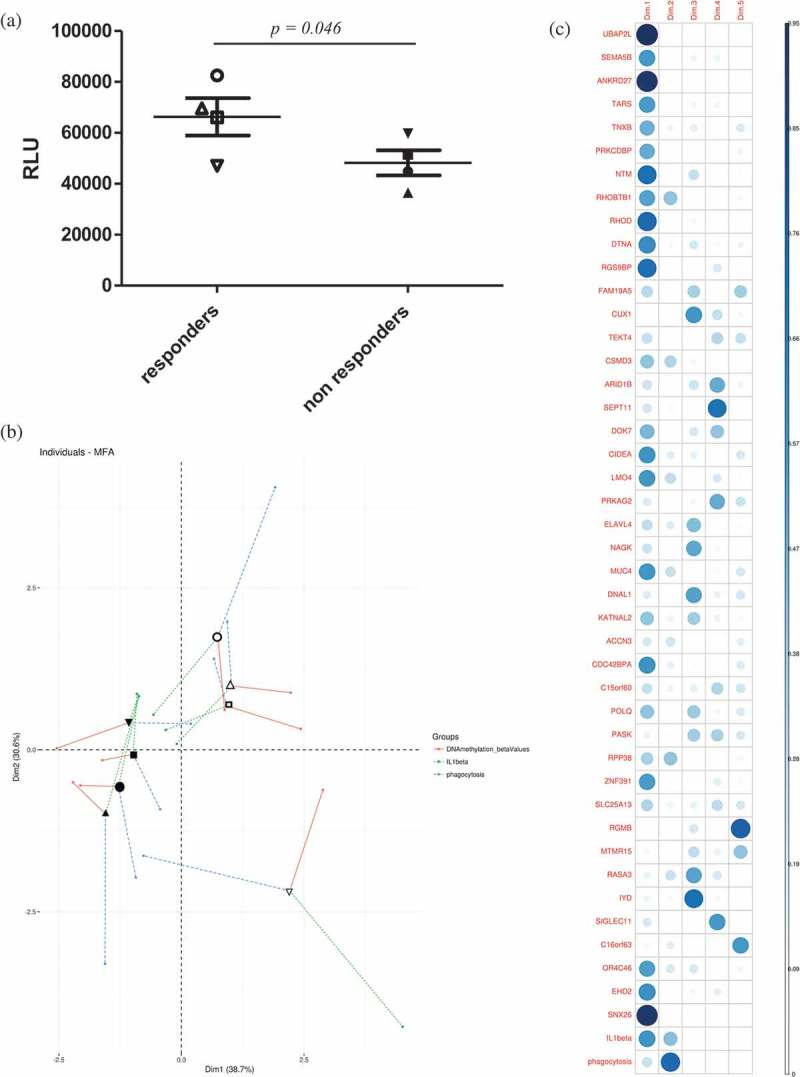
(a) Phagocytosis was measured as the number of luminescent virulent mycobacteria (arbitrary luminescence units) at 1 h post-infection of primary human macrophages. Empty symbols represent responders while the solid symbols represent the non-responders (two-tailed Student’s t-test, *p*-value = 0.046). (b) Individuals factor map obtained from the MFA using partial individual analysis. For a given individual, the symbol (as in Figure 5 (a)) corresponds to the centre of gravity for each individual, influenced by all groups of variables (the individual DMGs’ β values, phagocytosis and IL-1β, = 43+2 variables). (c) Correlation plot showed the 45 variables’ correlation distributions among five dimensions. The correlation values correspond to the quality of representations for variables on the factor map of MFA. The value was calculated as the squared coordinates, variance.cos2 = variance.coordinate × variance.coordinate. The circle size represents the value of variance.cos2 (correlation value) and the gradient of the colour scale shows the low cos2 (white) to high cos2 (blue).

The finding that the BCG responder-associated module and the miRNA-DMG network converged on pathways regulating actin remodelling, a process crucial for phagocytic uptake of bacteria, prompted us to more closely investigate the possible connection between the identified DMGs and phagocytosis and their subsequent impact on mycobacterial uptake [13]. We speculated that responders were more effective in initial uptake (= phagocytosis) of mycobacteria and analysed unpublished phagocytosis data from the previous study [7]. We found that the uptake of luciferase-expressing *M. tuberculosis* at day 0 (reflecting phagocytosis) was significantly more effective in responders as compared to non-responders (Figure 5(a)).

Finally, in order to further characterize the relationship between the tested parameters DNA methylation (the β value for each of the 43 DMGs), phagocytosis, and IL-1β, multiple factor analysis (MFA) was applied to the data collected for each subject. The mean representation of the samples according to the individual DMGs’ β-values, phagocytosis and IL-1β is presented in Figure 5(b) (individuals’ factor map, symbols reflecting each individual subject as in Figure 5(a)). The two groups (BCG responders and non-responders) can be distinguished in the plot depending on these 45 parameters. The level of IL-1β production in response to *M. tuberculosis* exposure, which we used as a starting point in the present study, was found to have the weakest contribution to the separation of the groups among the tested parameters. In a correlation plot derived from the MFA anlysis (Figure 5(c)), the contribution of the individual parameters (the individual DMGs, phagocytosis and IL-1β) is presented. Moreover, our data also highlighted that the DMGs UBAP2L (a ubiquitin-associated protein [29]), ANKRD27 (Ankyrin-repeat domain protein [30]) and RHOD (a Rho GTPase [31,32]) were the strongest determinants in distinguishing responders from non-responders.

## Discussion

This study expands on our previous observation of effective IL-1β production in mycobacteria-stimulated macrophages obtained from unvaccinated individuals, who later responded to BCG vaccination by reprogramming of DNA methylation and improving anti-mycobacterial efficacy [7]. Here, we investigated whether this propensity to respond to the vaccine was reflected in the DNA methylome of BCG responders already at the time point before vaccination. By comparing the DNA methylomes of immune cells obtained from BCG responders and non-responders two weeks before administration of the vaccine, we identified 43 DMGs that were differentially methylated. Exploring the functional relevance of those DMGs that had an annotated function together with their interacting partners, we identified a BCG responder-associated module with a majority of interacting proteins associated with the p75NTR pathway. This pathway is implicated in regulation of Rac [33] and Cdc42 [34]. Activation of Cdc42 and Rac results in the outgrowth of neurites in neurons [35] and actin reorganization causing membrane ruffling in non-phagocytic cells [36]. During phagocytosis, actin rearrangements are regulated via Cdc42 and Rac on one hand and Rho, on the other hand, inducing different routes of uptake via cup formation and a contractile mechanisms, respectively [37,38]. Of note, through a recently identified signalling mechanism, the mannose receptor, which is the primary receptor for mycobacterial uptake, also triggers Cdc42 and Rac activation [39]. Although the role of the p75NTR pathway has not been studied explicitly for phagocytic uptake, the circumstantial lines of evidence presented here suggest that this highly conserved pathway could be regulating phagocytosis when expressed in phagocytes. In fact, evidence exists that neurotrophin is regulating immune cell function [40]. In line with this, our analysis of experimental data reflecting uptake of mycobacteria showed that BCG responders’ macrophages were more efficiently phagocytosing the bacteria as predicted by the bioinformatic analyses of the DNA methylomes of responders and non-repsonders. The fact that phagocytosis correlated strongly with the individual β-values of the 43 DMGs, whereas IL-1β was weakly correlated with these parameters suggests that phagocytosis is a primary event regulated by the DMGs (and hence different between BCG responders and non-responders) and that IL-1β release is secondary to phagocytosis, not reflected in differential methylation. In fact, it is well established that IL-1β release is a defence mechanism towards intracellular pathogens that logically is preceded by phagocytosis of microbes or microbial invasion of cells. Therefore, a difference in phagocytic uptake can be reflected in different efficacy in IL-1β production.

**Figure 6.**
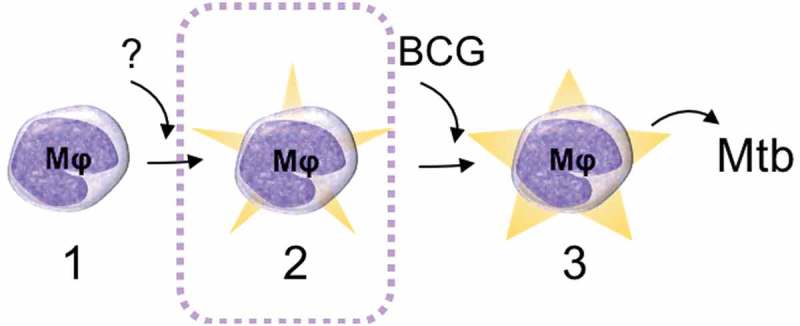
Schematic drawing of the proposed epigenetically different states of macrophages (Mφ). In the studied BCG-naïve population, two ‘states’ of immune cells could be identified: state 1 that does not respond effectively to the BCG vaccine (naïve state) and state 2 that has an increased capacity to ingest mycobacteria (primed state) and thereby respond efficiently to BCG by reprogramming the DNA methylome and enhance mycobactericidal effector mechanisms (the mycobactericidal state, 3). The mechanism behind the transition from state 1 to state 2 remains elusive. The transition from state 2 to state 3 can be induced through BCG vaccination.

We were able to validate the relevance of the identified DMGs for mycobacterial exposure by matching our findings with previous studies describing alterations in DNA methylation [19] and miRNA patterns [24,25] in response to mycobacterial challenge. Although the data analysed in the present study (derived from non-mycobacteria-exposed individuals) is not at a first glance expected to overlap with data derived from mycobacteria-exposed samples, possible explanations for this correlation exist. First, a previous exposure to mycobacteria (e.g. environmental mycobacteria) could evoke a transition from the ‘naïve state’ (state 1 in Figure 6) to the ‘primed state’ (state 2 in Figure 6). Second, it is possible that the same regions in the genome are targeted for methylation events both during the transition from the ‘naïve state’ to the ‘primed state’ (triggered by an unknown stimulus) and by mycobacterial stimulus (e.g. BCG vaccination) during the transition from the ‘primed state’ to the ‘mycobactericidal state’.

Importantly, as shown in our previous study [7], the ‘mycobactericidal state’ (state 3 in Figure 6) is also reflected in the DNA methylome but with mechanisms related to other immune functions such as interferon-γ production rather than phagocytosis.

The present study is based on observations made with a limited number of subjects and further studies are needed to corroborate the findings in larger cohorts. Many ongoing and finalized studies on modified BCG vaccine candidates have collected material that could be reanalysed with respect to epigenetic patterns like those observed here. If validated, the identified BCG responder module could be a valuable tool to predict who is a BCG responder/non-responder and identify interventions that could facilitate the transition of non-responders to BCG responders. In the long-term perspective, the results, therefore, could be used to guide the development of a new TB vaccine.

## Methods

### Data collection

#### DNA methylation dataset

In our previous study [7], Illumina HumanMehtylation450 (450K) DNA methylation data (GSE104287, https://www.ncbi.nlm.nih.gov/geo/query/acc.cgi?acc=GSE104287) was obtained from peripheral blood mononuclear cells (PBMCs) isolated from eight healthy subjects. The dataset obtained at the time point before BCG vaccination was subjected to a more thorough analysis in the present study. Based on the previous findings, four subjects were classified as ‘responders’ and the others as ‘non-responders’ based on their macrophages’ capacity to control experimental *M. tuberculosis* infection.

#### CpG hotspot data collection

To assess whether the identified differentially methylated genes (DMGs) were overlapping with the differentially methylated regions (DMRs) or not, we collected the data and annotations from a previous study by Sharma *et al.* [19] and compared with our dataset.

### Data processing and identification of differentially methylated genes

We processed the raw IDAT files of the Illumina DNA methylation data (GSE104287) with the minfi package(v1.4) using the quantile normalization and ChAMP (v2.8.9) package in R (v3.5) [41–43]. We computed the data by filtering those probes which failed to detect CG probes (no CG pair in the IDAT files, with a detection *p*-value < 0.01) [43,44] sites in the Illumina 450K array (removing 3,091 probes), probes with SNPs (removing 50,623 probes), potential cross-hybridized probes (removing 7,182 probes) and probes related to XY chromosomes (removing 10,410 probes) and with *p*-values < 0.05, finally resulting in a dataset including 414,206 probes. The β-values and M-values were calculated from the dataset and applied to determine the differential methylation with adjusted *p*-values < 0.05. The methylation status of genomic regions including transcription start sites at 201–1500 bp (TSS1500), transcription start sites at 1–200 (TSS200), 3ʹ untranslated regions (3ʹ-UTRs), 5ʹ- untranslated regions (5ʹ-UTRs) and 1^st^ exon and gene body regions were analysed. Using *p*-values with BH correction < 0.05, the probes allowed the annotation of 43 genes with differential methylation patterns comparing data obtained from responders with non-responders.

### Pathway enrichment analysis and gene–gene interaction networks

To measure the pathway enrichment, we used the gsePathway function of ReactomePA package with 1000 number of permutations, minimum GS size (minGSSize) = 120, and pAdjust method = ‘BH’. ModuleDiscoverer package [17] was applied to the dataset to determine the enriched gene module in the data. The interaction of the enriched module was calculated by using the default parameters and mapped to the STRINGdb package [18] (version = ”10”, score_threshold = 900, species = 9606) in R to annotate with the corresponding proteins connected.

Human gene–gene interaction data was collected from BioGRID database (v3.4) (https://thebiogrid.org) [14] and compared with our dataset to identify the interacting partners of these 43 differentially methylated genes (Figure S2).

### Interactions between MicroRNAs and differentially methylated genes

MicroRNA (miRNA) data were obtained from miRwalk v2.0 (http://zmf.umm.uni-heidelberg.de/apps/zmf/mirwalk2/) [45], TargetScan v7.1 (http://www.targetscan.org/vert_71/) [46], DIANA MicroT v5 (http://diana.imis.athena-innovation.gr/DianaTools/index.php?r=microT_CDS/index) [47], miRbase release 22, March 2018) [48] databases. MiRwalk, TarBase and microT database were selected as they were compiled based on the experimentally validated and manually curated miRNA-gene targets. TargetScan is known for its accuracy and also has a high fidelity of target prediction using the seed pairing mechanism between miRNA: mRNA. To increase the reliability of our data, we used only conserved binding with P_CT_ score ≥ 0.8. All miRNA-gene target interaction data were unified, redundancy removed and mapped to our dataset.

We obtained a list of 24 miRNAs that have been linked to mycobacterial exposure of human cells or tissues in previously published literature after comparing with miRNAs present in our dataset. The list was used in DIANA miRpath v3 (http://snf-515788.vm.okeanos.grnet.gr) with default parameter setup [49] for pathway enrichment using the KEGG database.

### Multiple factor analysis

Multiple factor analysis (MFA) was performed on the IL-1β, DNA methylation, and phagocytosis datasets using R packages FactoMineR [50] and factoextra [51]. MFA analysis was used as the data have quantitative variables and three different factors are considered. Quantitative variables in MFA were subjected to principal component analysis (PCA) and additionally, the different factors were normalized by assigning the same weight (based on the first eigen-value of the analysis) for the same group and differing from other groups. The representation of the variables was used to describe the dimensions as in PCA. The ellipses illustrated the 95% confidence interval of the data.

### Statistical calculations

All statistical analysis was performed in SPSS v24 and R v3.5. We used hypergeometric test to perform the functional enrichment analysis of the miRNAs. Bootstrap with 1,000,000 permutations was used to detect overlap enrichment analysis with regionR package [52] in R to identify the common regions in Sharma’s study. We used independent student’s t-test (two-tailed) to analyse the methylation difference between the responders and non-responders. The results were considered to be statistically significant when the *p*-value was < 0.05.

### Data validation

To validate the identified functional categories, we reanalysed the functional data obtained in the previous study [7] to assess phagocytic capacity in *M. tuberculosis*-infected macrophages. The bacterial load of macrophages, reflective of phagocytic capacity, was determined at day 0 as described in [7].

## Data Availability

The raw Illumina Infinium Human Methylation 450K bead chip data that support the findings of this study is available in NCBI GEO database (Accession number GSE104287; https://www.ncbi.nlm.nih.gov/geo/query/acc.cgi?acc=GSE104287).
