# Supplementary material for: Identification of DNA methylation patterns predisposing for an efficient response to BCG vaccination in healthy BCG-naïve subjects
Source: Epigenetics. 2019 Apr 22;14(6):589–601. doi: 10.1080/15592294.2019.1603963 (PMC6557603; doi:10.1080/15592294.2019.1603963)
Supplement: Supplemental Material [file kepi-14-06-1603963-s001.pdf]

Supplementary material

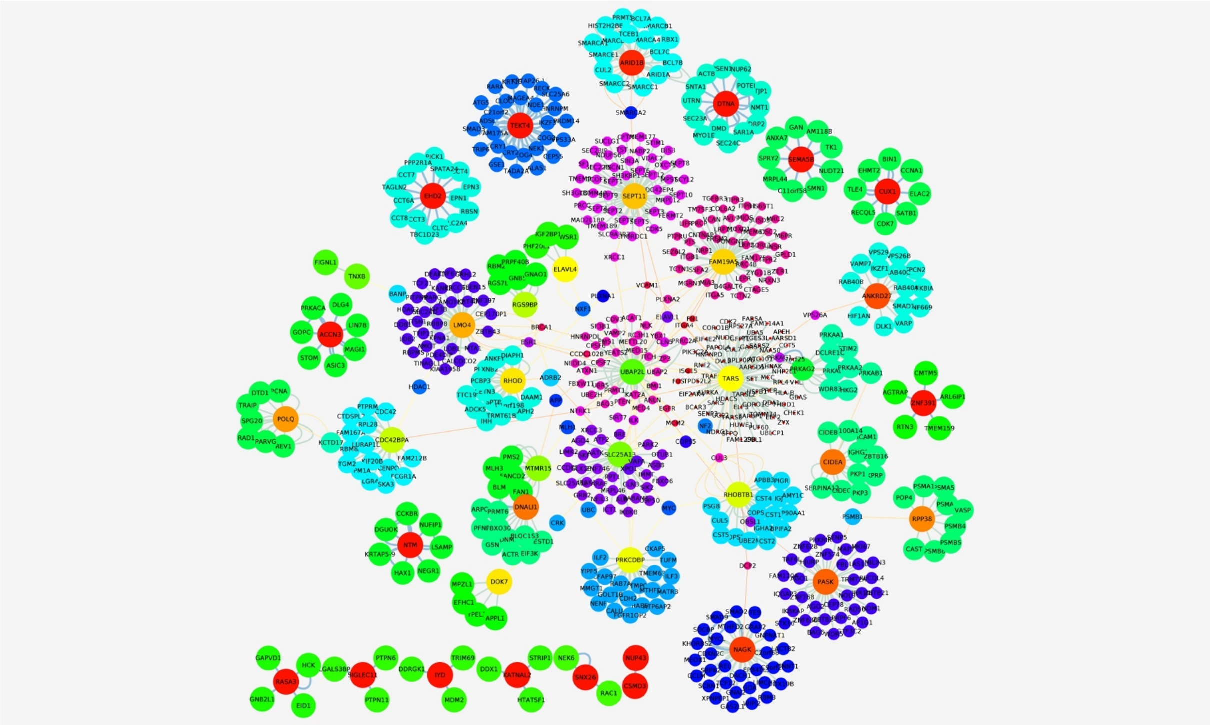

Figure S1

Interconnections among the 43 DMGs and their first interacting gene partners, identified from BioGRID database. The network was built in Cytoscape v3.5[13] using prefuse force directed layout on nodes and coloured the nodes in RGB using discrete mapping depending on the neighbourhood connectivity.

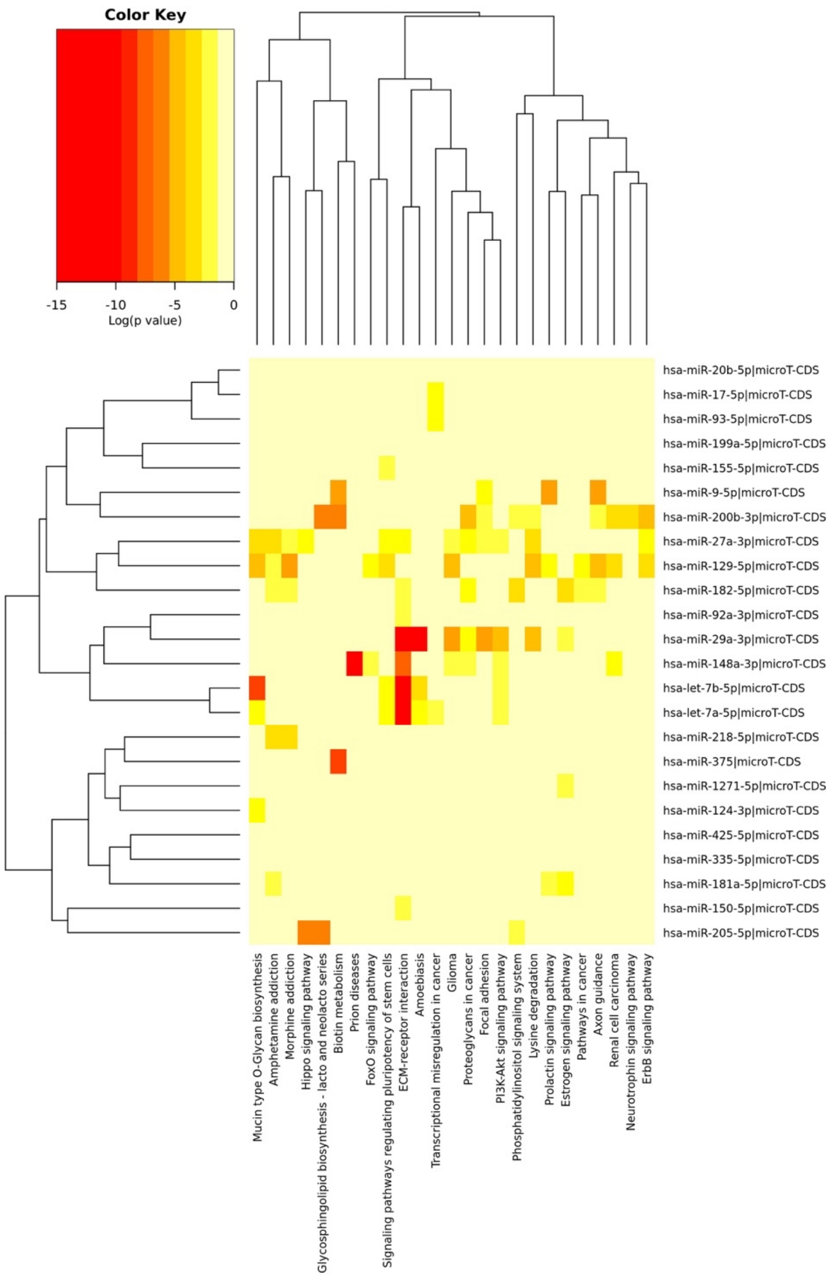

Figure S2

The heatmap shows the interactions among 24 miRNAs that target 35 DMGs and related to mycobacterial exposure of human cells and tissues and their enriched pathways in KEGG pathway analysis using DIANA miRpath v3.0. The analysis was done with the following parameter set up: *p*-value threshold < 0.05, MicroT threshold = 0.8, enrichment analysis method: Fisher's exact test (hypergeometric distribution), FDR corrected and conservative stats in pathway union.

Supplementary Tables

Supplementary Table 1

Chromosomal positions and features of the 43 DMGs. The Bonferroni-Hochberg (BH) corrected *p*-value reflects the stringency of the differential methylation comparing responders' and non-repsonders' DMGs. Chr: chromosome; bp: base pairs.

**DMG**	**Chromosomal positions**	**Start position (bp)**	**End position (bp)**	**Feature**	**Adjusted *p*-value (BH corrected)**
ANKRD27	chr19	33096468	33096704	5'UTR	3,574E-06
RGS9BP	chr19	33165266	33168099	3'UTR	7,148E-06
CDC42BPA	chr1	227505446	227506752	Body	1,07E-05
SEMA5B	chr3	122631722	122632854	5'UTR	1,43E-05
UBAP2L	chr1	154244710	154245289	Body	1,79E-05
EHD2	chr19	48216486	48216837	Body	2,14E-05
ARID1B	chr6	157342060	157343075	Body	2,50E-05
PRKAG2	chr7	151300169	151300726	5'UTR	2,86E-05
PRKCDBP	chr11	6340445	6341909	TSS200	3,22E-05
SNX26	chr19	36266234	36266622	TSS1500	3,57E-05
TARS	chr5	33440635	33441365	TSS1500	3,93E-05
TNXB	chr6	32046815	32047094	Body	4,65E-05
KATNAL2	chr18	44526866	44527137	Body	5,00E-05
RGMB	chr15	93588102	93588570	3'UTR	5,36E-05
TEKT4	chr2	95537196	95537810	1stExon	0,04988563
RASA3	chr13	114782674	114782883	Body	0,04988921
RPP38	chr10	15138965	15139851	Body	0,04989278
MUC4	chr3	195477711	195477983	Body	0,04989636
DOK7	chr4	3464799	3465440	Body	0,0499035
DTNA	chr18	32073444	32074292	TSS200	0,0499178
FAM19A5	chr22	48884884	48887043	Body	0,04992137
RHOBTB1	chr10	62703307	62704290	TSS1500	0,04992495
PASK	chr2	242088415	242090330	Body	0,04992852
SLC25A13	chr7	95950779	95951636	Body	0,04993209
CSMD3	chr8	114444826	114445085	TSS200	0,04993567
DNAL1	chr14	74111560	74111766	TSS1500	0,04993924
RHOD	chr11	66824118	66824621	3'UTR	0,04994282

SEPT11	chr4	76894929	77083113	Body	0,04994639
SIGLEC11	chr19	49889654	50048776	TSS1500	0,04994996
ZNF391	chr6	27356492	27357020	5'UTR	0,04995354
LMO4	chr1	87793802	87795142	5'UTR	0,04995711
CIDEA	chr18	12254147	12255089	Body	0,04996069
ELAVL4	chr1	50513644	50514320	Body	0,04996426
CUX1	chr7	101457382	101461030	Body	0,04996783
POLQ	chr3	121264469	121264943	3'UTR	0,04997141
C16orf63	chr16	15982245	15982672	TSS1500	0,04997498
OR4C46	chr11	50409147	54657953	TSS200	0,04997856
NAGK	chr2	71295160	71296002	Body	0,04998213
C15orf60	chr15	73735322	73735609	TSS200	0,0499857
ACCN3	chr7	150746002	150746381	Body	0,04998928
IYD	chr6	150269412	150843665	5'UTR	0,04999285
NTM	chr11	131560356	131560559	Body	0,04999643
MTMR15	chr15	31195673	31196392	Body	0,0499976

Supplementary Table 2

The enriched pathways identified from 24 miRNAs which are linked with 35 DMGs in our dataset and also related to mycobacterial exposure of human cells/tissues, using DIANA miRpath v3 database. Each pathway shows the number of interacting miRNAs and their miRNA-IDs.

KEGG pathway	*p*-value	Number of miRNAs	miRNA IDs
ECM-receptor interaction	<1E-325	8	hsa-let-7a-5p, hsa-let-7b-5p, hsa-miR-148a-3p, hsa-miR-150-5p, hsa-miR-182-5p, hsa-miR-27a-3p, hsa-miR-29a-3p, hsa-miR-92a-3p
Prion diseases	2,753353E-14	1	hsa-miR-148a-3p
Amoebiasis	1,20495E-07	3	hsa-let-7a-5p, hsa-let-7b-5p, hsa-miR-29a-3p
Glioma	2,35471E-07	4	hsa-miR-129-5p, hsa-miR-148a-3p, hsa-miR-27a-3p, hsa-miR-29a-3p
Proteoglycans in cancer	1,20205E-06	5	hsa-miR-148a-3p, hsa-miR-182-5p, hsa-miR-200b-3p, hsa-miR-27a-3p, hsa-miR-29a-3p
Biotin metabolism	4,02666E-05	3	hsa-miR-200b-3p, hsa-miR-375, hsa-miR-9-5p
Focal adhesion	9,83251E-05	4	hsa-miR-200b-3p, hsa-miR-27a-3p, hsa-miR-29a-3p, hsa-miR-9-5p
Mucin type O-Glycan biosynthesis	0,0001374215	4	hsa-let-7a-5p, hsa-let-7b-5p, hsa-miR-129-5p, hsa-miR-27a-3p
Axon guidance	0,0002882573	4	hsa-miR-129-5p, hsa-miR-182-5p, hsa-miR-200b-3p, hsa-miR-9-5p
Signaling pathways regulating pluripotency of stem cells	0,0003822668	5	hsa-let-7a-5p, hsa-let-7b-5p, hsa-miR-129-5p, hsa-miR-155-5p, hsa-miR-27a-3p
ErbB signaling pathway	0,0004050557	3	hsa-miR-129-5p, hsa-miR-200b-3p, hsa-miR-27a-3p
Renal cell carcinoma	0,0007404345	3	hsa-miR-129-5p, hsa-miR-148a-3p, hsa-miR-200b-3p
Lysine degradation	0,0009331838	4	hsa-miR-129-5p, hsa-miR-200b-3p, hsa-miR-27a-3p, hsa-miR-29a-3p
PI3K-Akt signaling pathway	0,001480909	5	hsa-let-7a-5p, hsa-let-7b-5p, hsa-miR-148a-3p, hsa-miR-27a-3p, hsa-miR-29a-3p
Glycosphingolipid biosynthesis - lacto and neolacto series	0,003386808	2	hsa-miR-200b-3p, hsa-miR-205-5p

Prolactin signaling pathway	0,007234362	3	hsa-miR-129-5p, hsa-miR-181a-5p, hsa-miR-9-5p
FoxO signaling pathway	0,007480471	2	hsa-miR-129-5p, hsa-miR-148a-3p
Neurotrophin signaling pathway	0,007930287	1	hsa-miR-200b-3p
Amphetamine addiction	0,009790839	5	hsa-miR-129-5p, hsa-miR-181a-5p, hsa-miR-182-5p, hsa-miR-218-5p, hsa-miR-27a-3p
Estrogen signaling pathway	0,01303374	4	hsa-miR-1271-5p, hsa-miR-181a-5p, hsa-miR-182-5p, hsa-miR-29a-3p
Transcriptional misregulation in cancer	0,01989882	3	hsa-let-7a-5p, hsa-miR-17-5p, hsa-miR-93-5p
Pathways in cancer	0,02084909	2	hsa-miR-129-5p, hsa-miR-182-5p
Morphine addiction	0,02267029	4	hsa-miR-129-5p, hsa-miR-182-5p, hsa-miR-218-5p, hsa-miR-27a-3p
Prostate cancer	0,03939864	2	hsa-miR-129-5p, hsa-miR-29a-3p
Hippo signaling pathway	0,04069946	2	hsa-miR-205-5p, hsa-miR-27a-3p
